# Supplementary material for: Acute leucocyte, muscle damage, and stress marker responses to high-intensity functional training
Source: PLoS One. 2020 Dec 3;15(12):e0243276. doi: 10.1371/journal.pone.0243276 (PMC7714345; doi:10.1371/journal.pone.0243276)
Supplement: S1 Data — Pre- to post-changes in outcomes of leucocytes, neutrophils, lymphocytes, monocytes, creatine kinase, lactate, cortisol and area under the curve of cortisol following a single ‘Cindy’ workout session (M ± SEM [SD]) *Significantly different from pre-ex (p < 0.05); ‡Significantly greater than post-30min and post-24h (p < 0.05); ≠Significantly different between EXP and NOV group (p < 0.05); $Significantly lower than post-ex only for NOV (p < 0.05); αSignificantly greater than post-30min (p < 0.05); &Significantly greater than pre-ex only for ALL and EXP (p < 0.05). (PDF) [file pone.0243276.s001.pdf]

Table 5. - Pre- to post-changes in outcomes of leucocytes, neutrophils, lymphocytes, monocytes, creatine kinase, lactate, cortisol and area under the curve of cortisol following a single 'Cindy' workout session (M ± SEM [SD])

Variables	Group	Pre-ex	Post-ex	Post-30min	Post-24h
Total WBC (x10^3/μL)	EXP	7.1 ± 0.4 [1.3]	12.6 ± 0.7 [2.5]*‡	7.2 ± 0.5 [1.7]	7.4 ± 0.4 [1.4]
	NOV	6.5 ± 0.3 [0.9]	10.7 ± 0.5 [1.6]*‡	6.5 ± 0.4 [1.3]	6.6 ± 0.4 [1.2]
	All	6.8 ± 0.2 [1.1]	11.8 ± 0.5 [2.3]*‡	6.9 ± 0.3 [1.6]	7.1 ± 0.3 [1.4]
Neutrophils (x10^3/μL)	EXP	3.3 ± 0.2 [0.7]	4.6 ± 0.4 [1.5]*‡	4.3 ± 0.3 [1.1]*	3.5 ± 0.4 [1.3]
	NOV	3.2 ± 0.2 [0.6]	4.4 ± 0.3 [0.9]*‡	3.5 ± 0.3 [1.0]$^{*\$}$	3.3 ± 0.3 [1.0]
	All	3.3 ± 0.1 [0.7]	4.5 ± 0.3 [1.3]*	3.9 ± 0.2 [1.1]*	3.4 ± 0.2 [1.2]
Lymphocytes (x10^3/μL)	EXP	3.0 ± 0.3 [0.9]$^{\alpha}$	6.6 ± 0.4 [1.5]$^{*‡\neq}$	2.2 ± 0.2 [0.8]	3.0 ± 0.3 [0.9]$^{\alpha}$
	NOV	2.5 ± 0.1 [0.5]$^{\alpha}$	5.0 ± 0.3 [0.9]*‡	2.2 ± 0.1 [0.3]	2.4 ± 0.1 [0.4]$^{\alpha}$
	All	2.8 ± 0.2 [0.8]$^{\alpha}$	5.9 ± 0.3 [1.5]*‡	2.2 ± 0.1 [0.6]	2.7 ± 0.2 [0.8]$^{\alpha}$
Monocytes (x10^3/μL)	EXP	0,485 ± 0.026 [0,093]	0,956 ± 0.047 [0,168]*‡	0,468 ± 0.027 [0,096]	0,589 ± 0.031 [0,112]*
	NOV	0,416 ± 0.059 [0,186]	0,828 ± 0.114 [0,361]*‡	0,522 ± 0.048 [0,153]	0,525 ± 0.049 [0,155]*
	All	0,455 ± 0.029 [0,142]	0,900 ± 0.056 [0,270]*‡	0,492 ± 0.026 [0,124]	0,561 ± 0.028 [0,133]*
CK (U.L-1)	EXP	167.8 ± 40.2 [114.8]	217.5 ± 47.1 [169.7]*	207.8 ± 43.1 [155.4]*	273.8 ± 50.4 [181.9]*
	NOV	184.1 ± 37.4 [118.4]	238.6 ± 49.7 [157.1]*	224.9 ± 44.1 [139.6]*	279.9 ± 57.9 [183.0]*
	All	174.9 ± 27.4 [131.3]	226.7 ± 33.6 [161.0]*	215.3 ± 30.4 [145.6]*	276.5 ± 37.2 [178.2]*
Lactate (mmol.l^{-1})	EXP	2.0 ± 0.2 [0.6]	14.3 ± 0.7 [2.5]$^{*\alpha}$	5.0 ± 0.3 [1.1]*	-
	NOV	1.8 ± 0.4 [1.2]	12.4 ± 1.6 [5.1]$^{*\alpha}$	4.3 ± 0.6 [1.9]*	-
	All	1.9 ± 0.2 [0.9]	13.5 ± 0.8 [3.9]$^{*\alpha}$	4.7 ± 0.3 [1.5]*	-
Cortisol (μg/dl)	EXP	15.4 ± 0.7 [2.4]	19.6 ± 0.9 [3.3]$^{\&\neq}$	19.9 ± 1.1[4.0]$^{*\neq}$	-
	NOV	13.4 ± 1.2 [3.7]	13.9 ± 1.1 [3.6]	15.9 ± 1.4 [4.5]*	-
	All	14.6 ± 0.7 [3.0]	17.2 ± 0.9 [4.3]$^{\&}$	18.2 ± 1.0 [4.4]*	-
Area under the curve of Cortisol (μg/dl)	EXP	-	-	943.9 ± 44.1 [159.2]$^{\neq}$	-
	NOV	-	-	720.9 ± 54.1 [171.2]	-

*Significantly different from pre-ex ($p < 0.05$);

‡Significantly greater than post-30min and post-24h ($p < 0.05$);
≠Significantly different between EXP and NOV group ($p < 0.05$);
$Significantly lower than post-ex only for NOV ($p < 0.05$);
αSignificantly greater than post-30min ($p < 0.05$).
&Significantly greater than pre-ex only for ALL and EXP ($p < 0.05$).
