# Supplementary material for: Acute leucocyte, muscle damage, and stress marker responses to high-intensity functional training
Source: PLoS One. 2020 Dec 3;15(12):e0243276. doi: 10.1371/journal.pone.0243276 (PMC7714345; doi:10.1371/journal.pone.0243276)
Supplement: Attachment — Submitted filename: Response to Reviewers.docx [file pone.0243276.s003.docx]

**Response to Reviewers**

**PONE-D-20-16542R1**

**Acute leucocyte, muscle damage, and stress marker responses to high-intensity functional training**

**Reviewer #1:**

**General comments:**

Authors have fully addressed my suggestions, and raised the quality of their manuscript. Very few studies on HIFT in the world have the quality of the present paper. Congratulations!

**Answer:** Dear reviewer, we are flattered by your words. It is extremely valuable for us to receive your congratulations. Your contributions were essential for us to reach this level of quality.

**Reviewer #2:**

**General comments:**

Congratulate the authors for the effort done in improving the paper and addressing my concerns. Nevertheless, I still think some issues need clarity.

**Answer:** Dear reviewer, we are very grateful for the considerations. It is a great honor for us to receive your compliments.

Discussion

P. 16, Line paragraph - 390 "We believe that the difference in VO2max is what mainly caused the EXP group to perform more rounds and maintain the same alterations in lactate concentration, as the maximum strength level revealed no differences between groups. Although the NOV group had only 3 to 8 months of experience with HIFT, they were all well versed in strength training."

This paragraph is not supported by scientific evidence. Butcher et al., (2015) concluded that CrossFit benchmark Cindy performance cannot be predicted by VO2. Perhaps the difference between EXP and NOV is explained by Local Muscle Endurance.

Butcher et al. Open Access J Sports Med. 2015 Jul 31;6:241-7. D

**Answer:** We agree with the observation and thank you for the indication of the article "Do physiological measures predict selected CrossFit (®) benchmark performance?”. Therefore, we reformulated the lines mentioned according to the information described in that article.

(*Line 392-406*)
